# Endothelial Cell CD36 Reduces Atherosclerosis and Controls Systemic Metabolism

**DOI:** 10.3389/fcvm.2021.768481

**Published:** 2021-11-23

**Authors:** Umar R. Rekhi, Mohamed Omar, Maria Alexiou, Cole Delyea, Linnet Immaraj, Shokrollah Elahi, Maria Febbraio

**Affiliations:** ^1^Department of Dentistry, University of Alberta, Edmonton, AB, Canada; ^2^Department of Medical Microbiology and Immunology, University of Alberta, Edmonton, AB, Canada

**Keywords:** CD36, fatty acid transport, endothelium, atherosclerosis, metabolism

## Abstract

High-fat Western diets contribute to tissue dysregulation of fatty acid and glucose intake, resulting in obesity and insulin resistance and their sequelae, including atherosclerosis. New therapies are desperately needed to interrupt this epidemic. The significant idea driving this research is that the understudied regulation of fatty acid entry into tissues at the endothelial cell (EC) interface can provide novel therapeutic targets that will greatly modify health outcomes and advance health-related knowledge. Dysfunctional endothelium, defined as activated, pro-inflammatory, and pro-thrombotic, is critical in atherosclerosis initiation, in modulating thrombotic events that could result in myocardial infarction and stroke, and is a hallmark of insulin resistance. Dyslipidemia from high-fat diets overwhelmingly contributes to the development of dysfunctional endothelium. CD36 acts as a receptor for pathological ligands generated by high-fat diets and in fatty acid uptake, and therefore, it may additionally contribute to EC dysfunction. We created EC CD36 knockout (CD36°) mice using cre-lox technology and a cre-promoter that does not eliminate CD36 in hematopoietic cells (Tie2e cre). These mice were studied on different diets, and crossed to the low density lipoprotein receptor (LDLR) knockout for atherosclerosis assessment. Our data show that EC CD36° and EC CD36°/LDLR° mice have metabolic changes suggestive of an uncompensated role for EC CD36 in fatty acid uptake. The mice lacking expression of EC CD36 had increased glucose clearance compared with controls when fed with multiple diets. EC CD36° male mice showed increased carbohydrate utilization and decreased energy expenditure by indirect calorimetry. Female EC CD36°/LDLR° mice have reduced atherosclerosis. Taken together, these data support a significant role for EC CD36 in systemic metabolism and reveal sex-specific impact on atherosclerosis and energy substrate use.

## Introduction

CD36 is a transmembrane glycoprotein, which was first described on platelets and later found to be expressed on an extensive range of cells and tissues including endothelial cells (ECs), monocytes/macrophages, adipocytes, hepatocytes, and muscles (1–5). CD36 plays a role in the uptake of apoptotic cells and modified lipoproteins, and in the recognition of ligands that trigger an innate immune response (6–11). It facilitates fatty acid (FA) transport into white and brown adipose tissue, heart and skeletal muscle, but the mechanism is not well-understood (12). Recent data also suggest a role in dermal albumin transcytosis, adipocyte FA export, and in tumor growth and metastasis (13–16). Structurally, CD36 has two transmembrane domains that terminate with very short intracellular domains (17). Despite these short cytoplasmic domains, work by our lab and others has shown that CD36 signals and is involved in cellular responses related to angiogenesis, innate immunity, scavenger receptor activity, EC apoptosis, cell migration, nuclear factor kappa beta, and inflammasome activation (10, 18–30). These responses affect the development of obesity, atherosclerosis, thrombosis, insulin resistance, and inflammation.

Metabolically active tissues, including heart, skeletal muscle and brown fat, require FA to meet their energy demands. While it is true that FA can diffuse across membranes (flip/flop mechanism), it has become apparent that uptake of long-chain FA, the major FA in our diet, is mediated by transporters, and this is necessary for efficient regulated supply to tissues and to prevent inappropriate delivery (31–34). FA uptake by CD36 has been controversial, both because protein-mediated FA uptake in general was thought to be unnecessary, and because CD36 does not structurally resemble a transporter (35). Considerable work in humans and animal models, however, supports a role for CD36 in FA transport (12, 36–43). The greatest bodily FA consumer is the heart, and CD36-deficient mice and humans have been shown to have decreased cardiac FA uptake and increased glucose uptake (12, 39, 40). In isolated working heart studies, global and cardiomyocyte-specific CD36° mice were shown to have a significantly lower rate of FA oxidation and a compensatory increase in glucose oxidation (44, 45).

We and others have shown that CD36 plays a role in obesity, atherosclerosis, and insulin resistance by both uptake of ligands and promotion of inflammatory signaling pathways, with the resultant secretion of reactive oxygen species and cytokines (29, 36, 46–49). This previous work was focused on macrophages, adipocytes, heart, and skeletal muscle. While global CD36 deficiency was protective against high-fat diet-induced obesity and insulin resistance, absence of CD36 in macrophages (using a bone marrow transplant approach) was not, in spite of reduction in macrophage inflammatory pathways (47). Both global and macrophage deficiency of CD36 were found to be protective against atherosclerosis (29, 48, 49).

Initiation of atherosclerosis has been shown to be a result of changes in EC, allowing prediction of atheroprone sites in the vasculature as a function of EC inflammation and dysfunction (50–52). Hyperlipidemia exerts pro-atherogenic decreased shear stress, and increased plasma FA and modified lipoprotein ligands trigger EC pro-inflammatory pathways, leading to expression of immune cell chemoattractants and surface receptors (52–57).

We hypothesize that CD36 is a major EC receptor for pro-inflammatory atherogenic ligands, and as such will regulate EC inflammation and have a significant impact on the initiation of atherosclerosis lesions. In this report, we show evidence to support the important role of EC CD36 in regulating systemic metabolism and in the development of atherosclerosis.

## Materials and Methods

### Experimental Animals and Diets

All animal procedures were prior approved by the University of Alberta Animal Care and Use Committee (AUP 0001953). To study the role of EC CD36 in metabolism and its effect on atherosclerosis, floxed (fl) *CD36* mice in the C57Bl/6j background were created by our lab by targeting exon 2 (exon 3 was included due to proximity), which contains the translation start site and first transmembrane domain, as previously described (44). These mice were then crossed to EC-specific Tie2e cre mice (58–60). Fl/fl *CD36* Tie2e cre+ mice were mated to LDLR knockout (LDLR°) mice (The Jackson Laboratory, B6.129S7-Ldlr^tm1Her^/J, IMSR Cat# JAX:002207, RRID:IMSR_JAX:002207), a classic atherosclerosis model, to generate fl/fl *CD36*/LDLR° and fl/fl *CD36* Tie2e cre+/LDLR° mice (EC CD36°/LDLR°). The mice were genotyped as previously described (44). EC CD36°/LDLR° and fl/fl *CD36*/LDLR° mice were fed with a diet containing 42.7 kilocalorie (kcal) % carbohydrate, 42 kcal% fat, and 1.25% added cholesterol (TD 96121, Envigo) for 3, 6, and 16 weeks for atherosclerosis studies (different cohorts). This diet was used previously in CD36°/LDLR° studies to induce CD36-dependent atherosclerosis (29, 30, 61). For metabolic analyses, mice were fed with ingredient-matched diets containing 35 kcal% carbohydrate and 45 kcal% fat (D12451, Research Diets, Inc.) or 70 kcal% carbohydrate and 10 kcal% fat (D12450H, Research Diets, Inc.); the amount of sucrose is the same.

### *En face* Aortic Morphometric Analysis

Dissection, processing, and *en face* morphometry of aortas were completed as previously described (29, 30, 49, 61). Briefly, the mice were euthanized by pentobarbital overdose (200 mg/kg, intraperitoneal) and the vasculature perfused through the heart with 10 ml phosphate buffered saline followed by 5 ml buffered formalin (Formaldefresh, Fisher Scientific). The entire aorta, from the heart, including the subclavian, right, and left carotid arteries, and extending 2–5 mm after bifurcation of the iliacs, was dissected free of fat and postfixed in buffered formalin for 24 h at 4°C and then stored in phosphate buffered saline at 4°C. Aortae were stained with oil red O (Sigma-Aldrich), which identifies neutral lipids in plaque, as per the manufacturer's instructions, to quantify lesion burden. After staining, the aortae were opened, laid flat, and digitally scanned. *En face* morphometry was performed in a blinded fashion. Briefly, three independent measurements of lesion area (red pixels) were selected and averaged for each aorta, using Adobe Photoshop software (RRID:SCR_014199). Similarly, the total aorta area was determined. The lesion area was expressed as mean percent ± S.E.M. of total aortic area.

### Glucose Tolerance Testing

For glucose tolerance testing, the mice were fasted overnight and received an intraperitoneal injection of 2 mg glucose per gram of body weight. Blood was drawn from the tail vein at baseline and then 15, 30, 45, 60, and 120 min after the administration of glucose. Blood glucose was measured using an Accu-Chek Advantage Glucometer. Area under the curve was calculated for individual curves using Graph Pad Prism software (RRID:SCR_002798).

### Total Cholesterol, Free Cholesterol, and Triglyceride Assays

The mice were fasted overnight and blood was collected by cardiac puncture into ethylenediaminetetraacetic acid containing syringes. The blood was centrifuged at 3,800 × g for 5 min and plasma aliquoted and frozen at −20°C. Total and free plasma cholesterol and triglyceride concentrations were measured using colorimetric assays (Wako chemicals, Fujifilm, Japan) as per the manufacturer's instructions. The levels in samples were determined by comparison against a curve constructed from serially diluted standards.

### Lipoprotein Analysis

The mice were fasted overnight and fresh plasma aliquots were used to perform fast protein liquid chromatography by the Lipidomics Core facility at the University of Alberta. The area under each peak was calculated using GraphPad Prism software (RRID:SCR_002798).

### Indirect Calorimetry

Four-week-old fl/fl *CD36* and EC CD36° mice were housed and maintained in metabolic cages. Following a 24-h acclimatization period, the mice were monitored over a 12 h light:12 h dark cycle (0600–1800 light) with *ad libitum* access to food (normal chow) and water. Indirect calorimetry was performed using the Comprehensive Laboratory Animal Monitoring System (Columbus Instruments). The respiratory exchange ratio (RER), calculated as the ratio of carbon dioxide to oxygen production, was used to calculate the percent contribution of fat (RER = 0.7) and carbohydrates (RER = 1) to whole body energy metabolism. Total activity of the mice was calculated by adding *Z* counts (rearing or jumping) to total counts associated with stereotypical behavior (grooming and scratching) and ambulatory movement. Data were analyzed using the web-based tool CalR (RRID:SCR_015849) (62).

### Flow Cytometry

Flow cytometry was performed on mouse blood as previously described (63). Briefly, to obtain single-cell suspensions, spleen samples were ground between sterile frosted glass slides in 7 ml of red blood cell lysis buffer (0.15 mM NH_4_Cl, 10 mM KHCO3, 0.1 mM disodium ethylenediaminetetraacetic acid, pH 7.2) and then filtered through sterile nylon mesh. Whole blood, obtained by cardiac puncture, was treated with red blood cell lysis buffer twice. Cell pellets were washed and resuspended in phosphate-buffered saline containing 2 mM ethylenediaminetetraacetic acid and 0.5% bovine serum albumin. Fluorophore-conjugated antibodies with specificity to mouse cell antigens were as follows: anti-CD11b (M1/70) (BD Biosciences Cat# 550993, RRID:AB_394002), anti-CD11c (HL3) (BD Biosciences Cat# 561022, RRID:AB_2033997), anti-SiglecF (E50-2440) (BD Biosciences Cat# 552125, RRID:AB_394340), anti-Ly6G (1A8) (BD Biosciences Cat# 561236, RRID:AB_10611860), and anti-CD36 (JC63.1) (Cayman Chemical Cat# 10009870, RRID:AB_10342682). Live/dead fixable dead cell stains (ThermoFisher) were used to exclude dead cells. CD11b+/c+ cells were analyzed for CD36 expression. Paraformaldehyde-fixed cells were acquired using a Becton Dickinson (BD) LSR Fortessa flow cytometer (BD Biosciences, University of Alberta Flow Cytometry core) and analyzed with FlowJo (version 10) software (RRID:SCR_008520).

### Dual-Energy X-Ray Absorptiometry

Lean and fat body mass were determined using dual-energy X-ray absorptiometry (Faxitron, model DXA UltraFocus, Hologic), as per the manufacturer's instructions. Acquisition, image processing, and analysis were performed by the associated Faxitron Vision software (Faxitron Bioptics LLC.).

### Statistical Analyses

Data are presented as mean ± S.E.M. Data were assessed for normal distribution by Shapiro–Wilk test. If normally distributed, the significance was evaluated using parametric Student's *t*-test. For non-normally distributed data, the significance was evaluated using Mann-Whitney. The significance was set at *p* < 0.05. Analyses and graphs were performed using GraphPad Prism software (RRID:SCR_002798).

## Results

**Figure 1:**
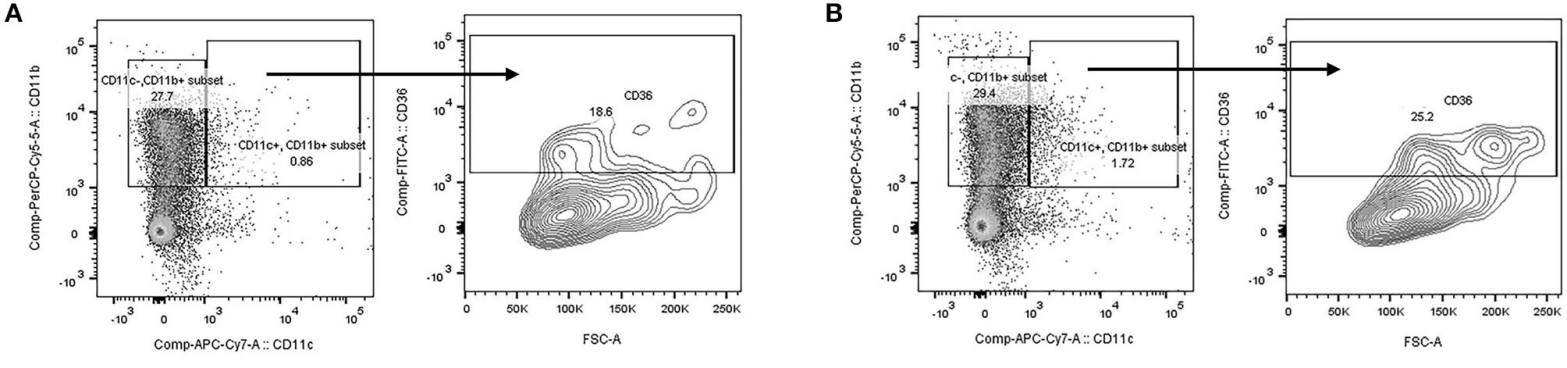
Flow cytometry analysis of mouse blood. Blood was prepared for flow cytometry and cells gated on the CD11b+/c+ population. CD36 expression was similar in **(A)** fl/fl *CD36* and **(B)** EC CD36° samples, demonstrating no loss of expression of CD36 on the monocyte population.

To demonstrate the specificity of cre expression, we performed flow cytometry on blood and gated on the CD11b+/c+ population. As shown in Figure 1, there was no loss of CD36 expression in EC CD36° samples (Figure 1B) compared with fl/fl *CD36* controls (Figure 1A). These data are in agreement with previous reports using this cre driver (58–60).

**Figure 2:**
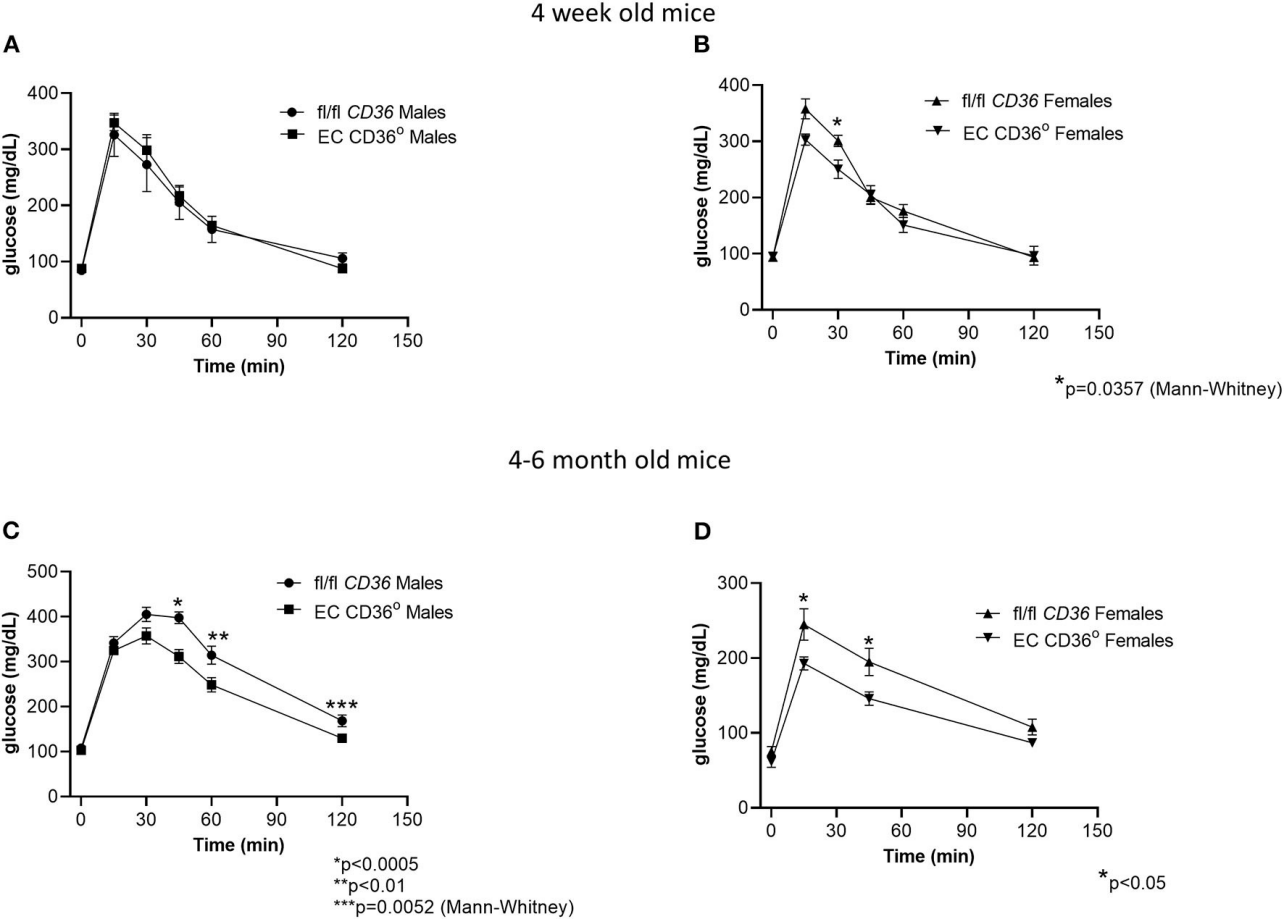
Glucose tolerance testing of normal chow-fed EC CD36° and fl/fl *CD36* mice. **(A)** Four-week-old male mice showed no differences (fl/fl *CD36, n* = 3; EC CD36°, *n* = 6). **(B)** Four-week-old female mice showed a significant difference at 30 min; area under the curve (AUC) was not significant (fl/fl *CD36, n* = 5; EC CD36°, *n* = 3). **(C)** Four–six-month-old male mice showed significant differences at 45, 60, and 120 min (fl/fl *CD36, n* = 13; EC CD36°, *n* = 12). AUC was significantly different (fl/fl *CD36*: 34,817 ± 1,437; EC CD36°: 28,888 ± 1,015, *p* < 0.005). **(D)** Four-six-month-old female mice showed significant differences at 15 and 45 min (*n* = 8/group). AUC was significantly different (fl/fl *CD36*: 20,341 ± 1,491; EC CD36°: 15,713 ± 862, *p* < 0.02).

To determine the impact of the loss of EC CD36 on basal systemic metabolism, we began with young mice that were similar in weight and lean body mass as measured by dual-energy X-ray absorptiometry. The mean percent lean body mass in 4-week-old fl/fl *CD36* male mice was 92.84 ± 0.26 vs. 93.54 ± 1.27 for EC CD36° males. Female fl/fl *CD36* mice had a mean lean body mass percent of 93.41 ± 0.37 vs. 94.56 ± 0.41 for EC CD36° females. Four-week-old EC CD36° male and female mice showed similar clearance of glucose compared with sex-matched controls in glucose tolerance testing (Figures 2A,B). At 4-6 months of age, both male and female EC CD36° showed significantly better glucose clearance compared with controls (Figures 2C,D), despite the similar weight.

**Figure 3:**
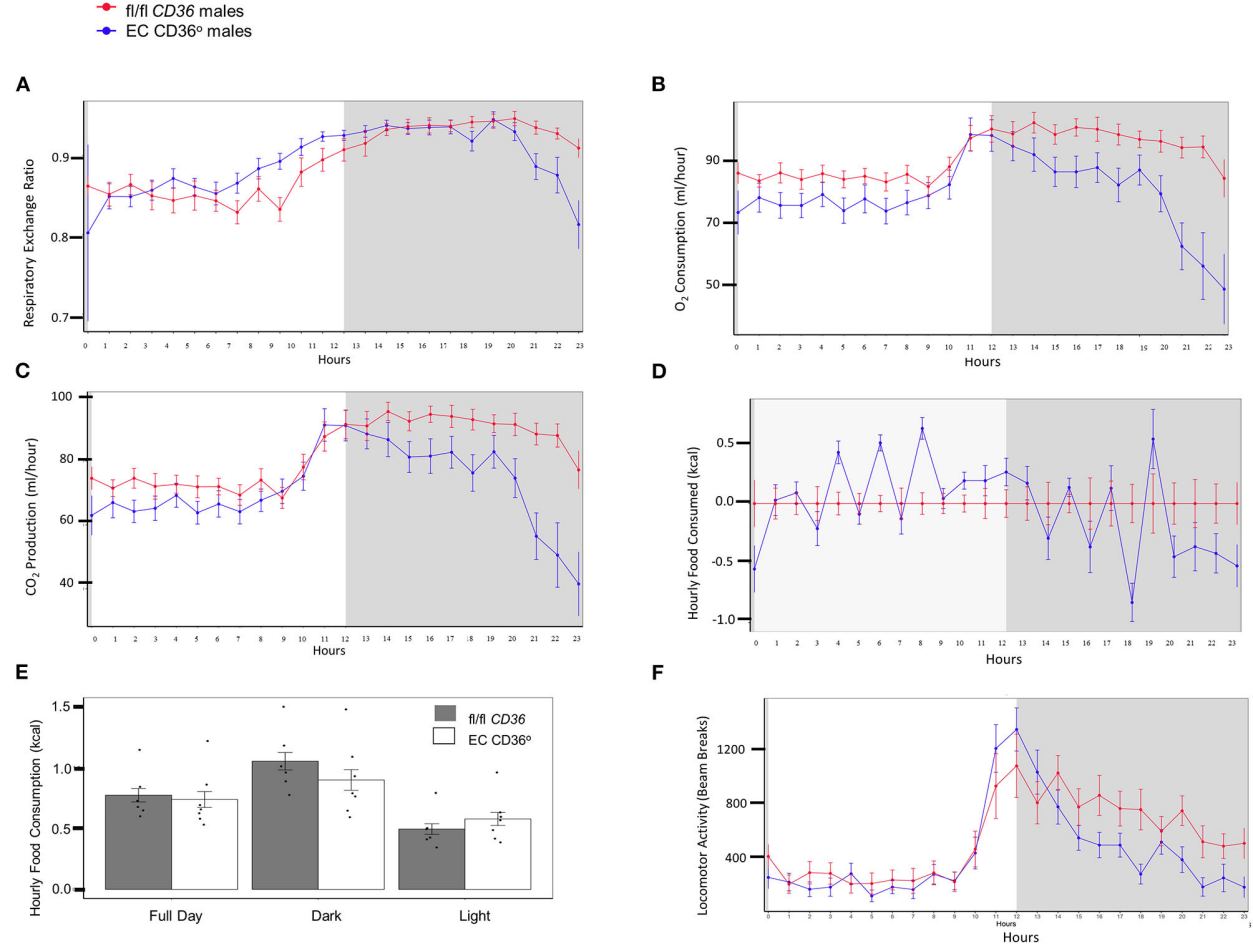
Indirect calorimetry of 4-week-old normal chow-fed male fl/fl *CD36* (*n* = 6) and EC CD36° (*n* = 7) mice. Plots show data from a 12 h light:12 h dark cycle after a 24 h acclimation period. White panel = light phase; gray panel = dark phase. **(A)** Respiratory exchange ratio, **(B)** oxygen consumption, **(C)** CO_2_ production, **(D)** food consumption, **(E)** total food consumption, and **(F)** locomotor activity.

Indirect calorimetry of 4-week-old normal chow-fed EC CD36° and controls showed mixed carbohydrate and fat use early in the light period for both strains of male mice (Figure 3A, Respiratory Exchange Ratio). EC CD36° males showed generally lower levels of oxygen consumption (Figure 3B) and carbon dioxide production (Figure 3C), indicative of lower energy expenditure, compared with controls. While cumulative food intake did not differ (Figures 3D,E), the time of feeding did. EC CD36° male mice ate more during the light period (Figures 3D,E), and this was reflected in increased carbohydrate metabolism after the midpoint of the light period (Figure 3A). The increase in carbohydrate usage by EC CD36° males continued until late in the dark period. EC CD36° males showed noticeably less locomotor activity (Figure 3F). Female mice did not show any differences in any parameters measured (data not shown).

**Figure 4:**
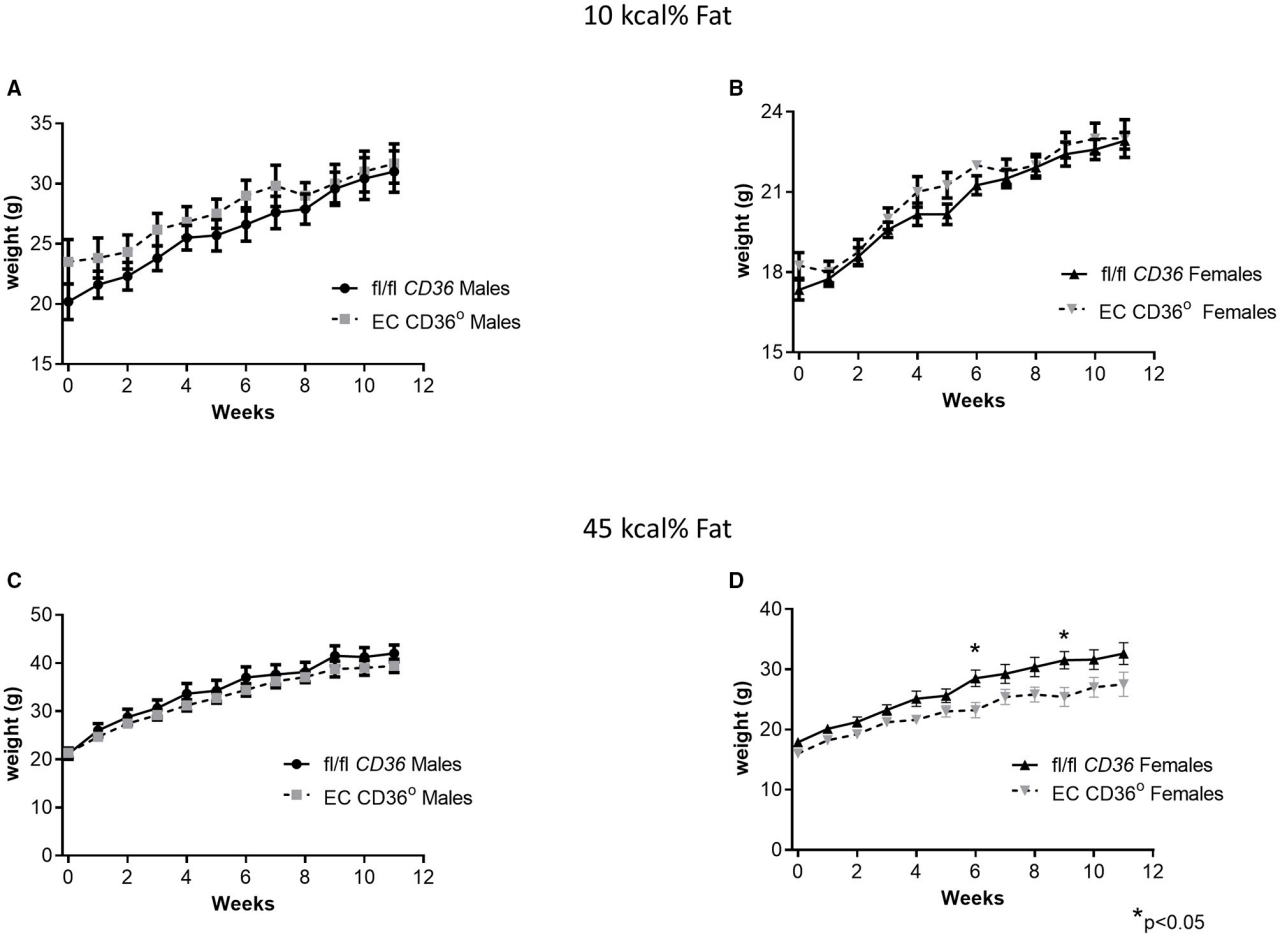
Change in weight over time of EC CD36° and fl/fl *CD36* mice fed with ingredient-matched diets. **(A)** Male mice fed with the 10 kcal% fat diet. (fl/fl *CD36, n* = 10; EC CD36°, *n* = 6). **(B)** Female mice fed with the 10 kcal% fat diet (fl/fl *CD36, n* = 12; EC CD36°, *n* = 4). **(C)** Male mice fed with the 45 kcal% fat diet. (fl/fl *CD36, n* = 8; EC CD36°, *n* = 9). **(D)** Female mice fed with the 45 kcal% fat diet (fl/fl *CD36, n* = 12; EC CD36°, *n* = 5). There was a significant difference in weight at the 6 and 9 week time points.

**Figure 5:**
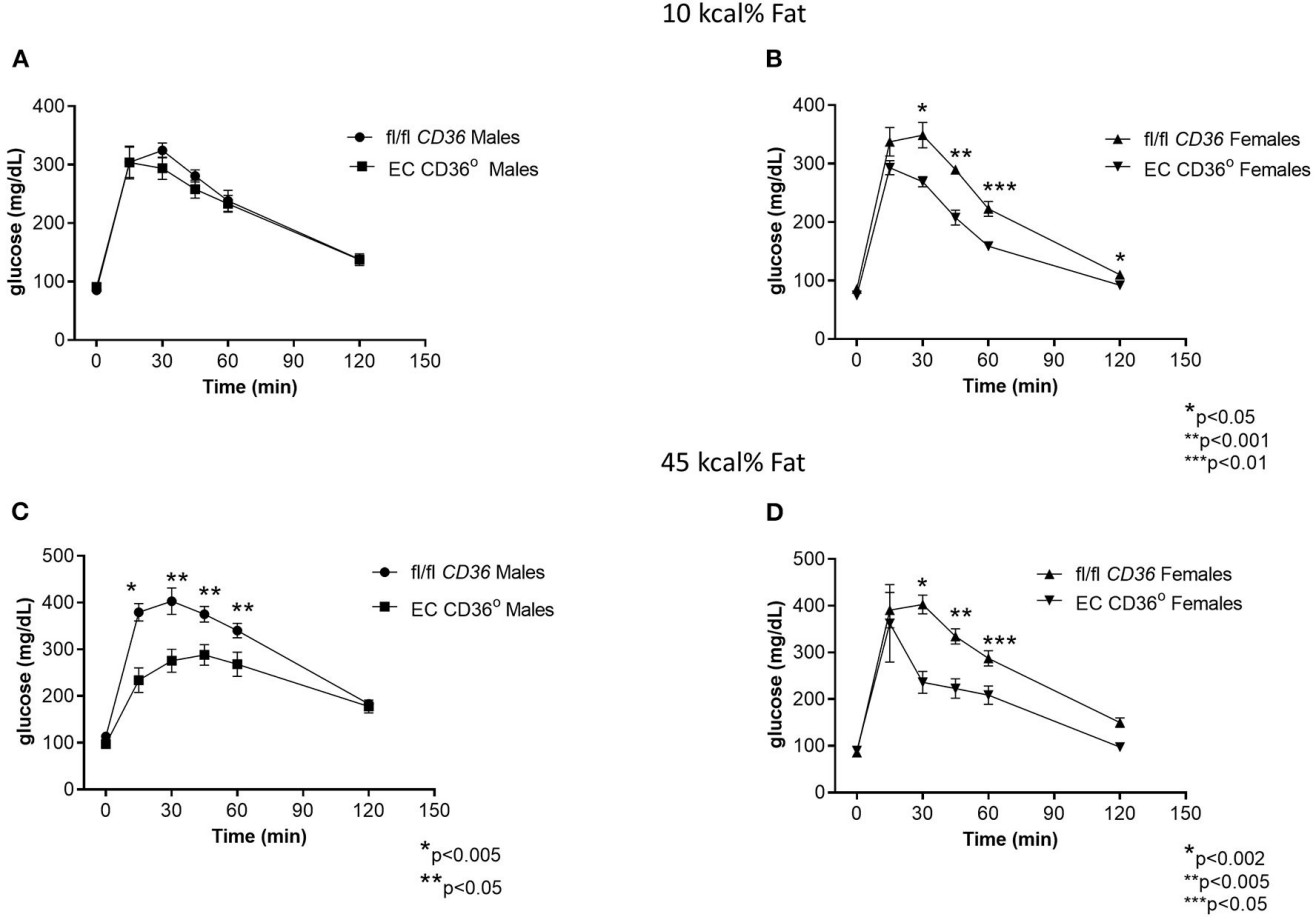
Glucose tolerance testing of EC CD36° and fl/fl *CD36* mice after 8 weeks feeding ingredient matched diets. **(A)** Male mice fed with the 10 kcal% fat diet showed no significant differences (fl/fl *CD36, n* = 5; EC CD36°, *n* = 6). **(B)** Female mice fed with the 10 kcal% fat diet showed significant differences at 30, 45, 60, and 120 min. AUC was significantly different (fl/fl *CD36*: 26,939 ± 688; EC CD36°: 20,815 ± 625, *p* = 0.001) (fl/fl *CD36, n* = 5; EC CD36°, *n* = 3). **(C)** Male mice fed with the 45 kcal% fat diet showed significant differences at 15, 30, 45, and 60 min. AUC was significantly different (fl/fl *CD36*: 36,455 ± 1,589; EC CD36°: 28,080 ± 1,974, *p* < 0.02) (fl/fl *CD36, n* = 5; EC CD36°, *n* = 3). **(D)** Female mice fed with the 45 kcal% fat diet showed significant differences at 30, 45, and 60 min. AUC was significantly different (fl/fl *CD36*: 32,822 ± 1,813; EC CD36°: 23,733 ± 62,135, *p* = 0.0238, Mann–Whitney) (fl/fl *CD36, n* = 5; EC CD36°, *n* = 3).

To determine the impact of the loss of EC CD36 in a diabetogenic setting, we fed the mice with ingredient-matched diets containing 35 kcal% carbohydrate and 45 kcal% fat (D12451, Research Diets, Inc.) or 70 kcal% carbohydrate and 10 kcal% fat (D12450H, Research Diets, Inc.); the extra carbohydrate is delivered in the form of starch, and the amount of sucrose between the diets is the same. At baseline, there were no differences in weight between the mice of the same sex. Weight gain was similar between the groups for both males and females over the 12 weeks of the diet, with transient small differences between the female groups at 6 and 9 weeks (Figures 4A–D). After 8 weeks of diet feeding, we compared glucose clearance by glucose tolerance testing (Figures 5A–D). Female EC CD36° mice showed better glucose clearance compared with controls when fed with either diet (Figures 5B,D); male EC CD36° mice showed improved glucose clearance compared with controls when fed with the 45 kcal% diet (Figure 5C). Lipoprotein analysis showed that the distribution of cholesterol was similar between the groups of both sexes when fed with either diet (Table 1). The distribution of triacylglycerides, however, showed differences, especially in female mice (Table 1). Female EC CD36° mice fed with the 10 kcal% fat diet had a greater percentage of triacylglycerides in the LDL/IDL (intermediate density lipoprotein) fraction compared with controls. Male and female EC CD36° mice fed with the 45 kcal% diet both showed a greater percentage of triaclyglycerides in the LDL/IDL fractions (Table 1) compared with controls. Female EC CD36° mice showed less triacylglycerides overall.

**Table 1:** Lipoprotein Analysis of Mice Fed With the 10 and 45 kcal % Fat Diets.

	**fl/fl** ***CD36***	**EC CD36^°^**	**fl/fl *CD36***	**EC CD36^°^**
**10 kcal% fat**	**Cholesterol (% of total)**		**Triacylglyceride (% of total)**	
**Males**				
VLDL	2	1	62	69
LDL/IDL	9	11	30	26
HDL	89	88	9	5
**Females**				
VLDL	2	2	51	41
LDL/IDL	7	11	39	48
HDL	91	87	11	11
**45 kcal% fat**	**Cholesterol (% of total)**		**Triacylglyceride (% of total)**	
**Males**				
VLDL	0.3	0.7	54	37
LDL/IDL	20	14	39	48
HDL	80	86	7	15
**Females**				
VLDL	0.8	0.5	27	25
LDL/IDL	11	9	46	64
HDL	88	91	27	12

**Figure 6:**
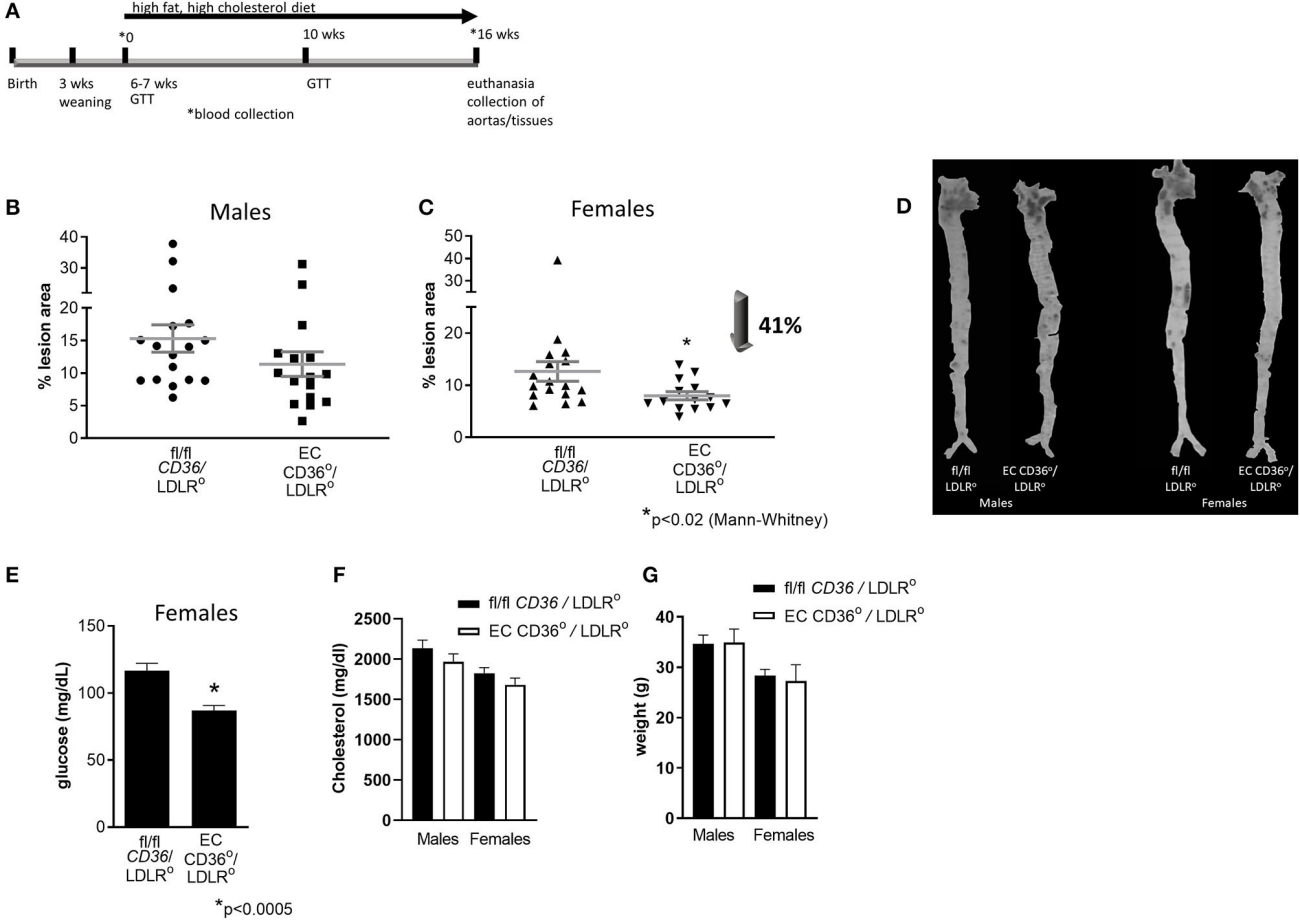
Atherosclerosis lesion analysis. *En face* oil red O aortic lesions were quantified using Adobe Photoshop software after 16 weeks high-fat high-cholesterol diet feeding. **(A)** Timeline of atherosclerosis study. This was a longitudinal study. Mice were weaned at 3 weeks of age, and the high-fat, high-cholesterol diet was begun when the mice were 6–7 weeks of age. Just prior to the diet start (0), glucose tolerance testing (GTT) was performed and blood was collected; this and other blood collection timepoints are indicated by *. GTT was also performed after 10 weeks of diet feeding. Mice were euthanized and aortas, blood, and tissues were collected after 16 weeks of diet feeding. **(B)** Male mice showed no difference in percent atherosclerosis lesion (fl/fl *CD36*/LDLR°: 15.31 ± 2.096% vs. EC CD36°/LDLR°: 11.37 ± 1.884%) (fl/fl *CD36*/LDLR°, *n* = 17; EC CD36°/LDLR°, *n* = 16). **(C)** Female EC CD36°/LDLR° mice showed a significant 40% decrease in percent atherosclerosis lesion compared with controls (fl/fl *CD36*/LDLR°: 12.65 ± 1.896% vs. EC CD36°/LDLR°: 7.447 ± 0.897%, *p* < 0.02, Mann–Whitney) (fl/fl *CD36*/LDLR°, *n* = 17; EC CD36°/LDLR°, *n* = 14). **(D)** Representative aortas stained with oil red O. **(E)** Female EC CD36°/LDLR° mice had significantly lower fasting glucose compared with controls. **(F)** Total cholesterol showed no differences in males (fl/fl *CD36*/LDLR°: 2,132 ± 103.4 mg/dL vs. EC CD36°/LDLR°: 1,967 ± 96.42 mg/dL) or females (fl/fl *CD36*/LDLR°: 1,824 ± 68.94 mg/dL vs. EC CD36°/LDLR°: 1,680 ± 84.2 mg/dL) at endpoint. **(G)** Weights were not different at endpoint in males (fl/fl *CD36*/LDLR°: 35.51 ± 1.34 g vs. EC CD36°/LDLR°: 34.88 ± 0.673 g) or females (fl/fl *CD36*/LDLR°: 28.6 ± 0.78 g vs. EC CD36°/LDLR°: 27.27 ± 0.831 g).

To determine the impact of the loss of EC CD36 on atherosclerosis, we crossed EC CD36° mice with the atherogenic LDLR° strain. We fed these mice a Western-style diet containing 42.7 kcal% carbohydrate, 42 kcal% fat, and 1.25% added cholesterol (TD 96121, Envigo) to promote atherosclerosis development, for 16 weeks. Figure 6A gives a general overview of the study timeline. *En face* morphometry of whole aortas stained with oil red O showed no difference in male mice (Figure 6B, *p* = 0.0872, Mann–Whitney). In contrast, female EC CD36°/LDLR° mice showed a 41% reduction in aortic lesions (Figure 6C). Representative aortas are shown in Figure 6D. Female mice showed decreased fasting glucose (Figure 6E). At sacrifice, there were no differences in plasma total cholesterol or weight in males or females (Figures 6F,G).

**Figure 7:**
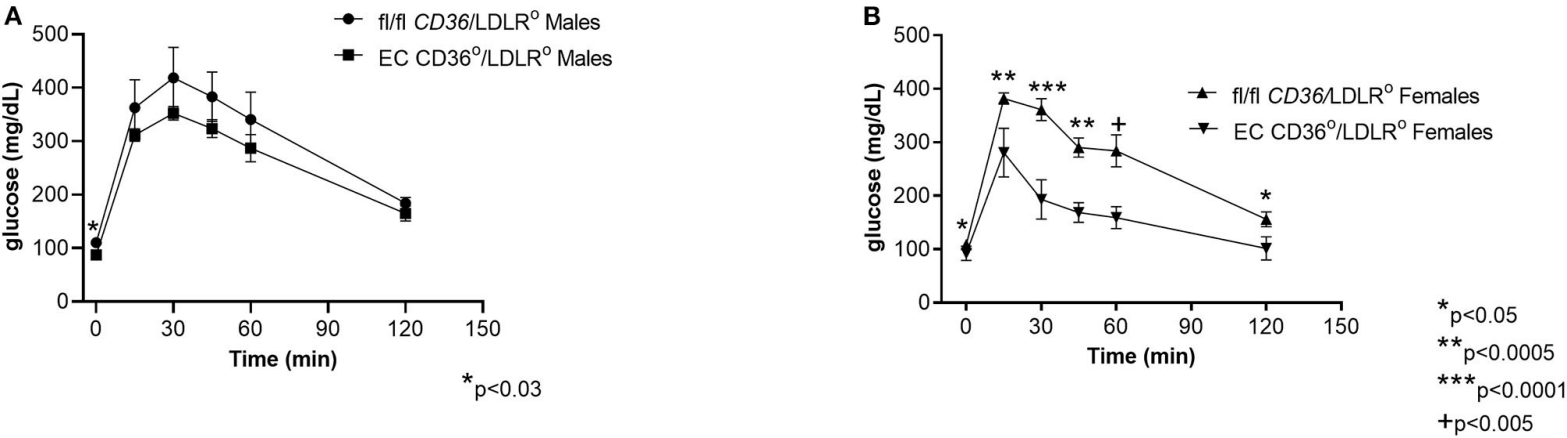
Glucose tolerance testing of EC CD36°/LDLR° and fl/fl *CD36*/LDLR° mice after 10 weeks of high-fat high-cholesterol diet feeding. **(A)** Male EC CD36°/LDLR° mice showed a significant difference at the 0 time point. AUC was not different (fl/fl *CD36*/LDLR°, *n* = 4; EC CD36°/LDLR°, *n* = 7). **(B)** Female EC CD36°/LDLR° mice showed significant differences at all time points. AUC was significantly different (fl/fl *CD36*/LDLR°: 30,817 ± 1,142; EC CD36°/LDLR°: 19,331 ± 1,161, *p* < 0.0001) (fl/fl *CD36*/LDLR°, *n* = 9; EC CD36°/LDLR°, *n* = 5).

**Figure 8:**
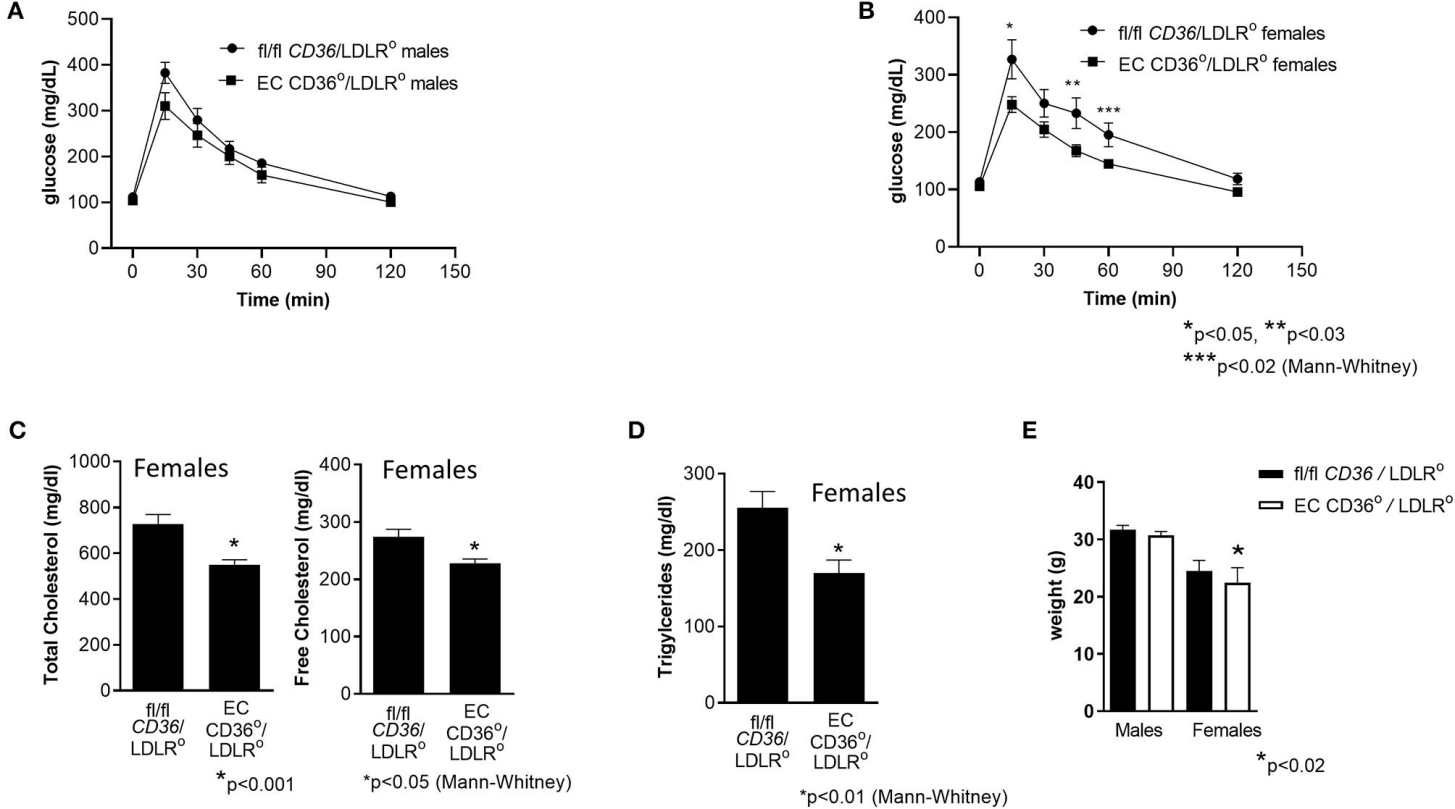
Glucose tolerance testing, plasma cholesterol, and triacylglycerides of female EC CD36°/LDLR° and fl/fl *CD36*/LDLR° mice at baseline and after 3 weeks high-fat high-cholesterol diet feeding. **(A)** At 6–7 weeks of age, prior to initiation of the high fat high cholesterol diet, there were no differences in glucose clearance between the groups. **(B)** After 3 weeks diet feeding, female EC CD36°/LDLR° showed significant differences at 15, 45, and 60 min time points. AUC was significantly different (fl/fl *CD36*/LDLR°: 23,896 ± 1,920; EC CD36°/LDLR°: 18,409 ± 719, *p* < 0.02) (fl/fl *CD36*/LDLR°, *n* = 8; EC CD36°/LDLR°, *n* = 10). **(C)** Plasma total and free cholesterol were significantly lower in EC CD36°/LDLR° female mice after 3 weeks diet feeding (fl/fl *CD36*/LDLR°, *n* = 10; EC CD36°/LDLR°, *n* = 13). **(D)** Plasma triacylglycerides were significantly lower in EC CD36°/LDLR° female mice after 3 weeks diet feeding (fl/fl *CD36*/LDLR°, *n* = 10; EC CD36°/LDLR°, *n* = 13). **(E)** There was no difference in weights at 8 weeks in males (fl/fl *CD36*/LDLR°: 31.68 ± 0.769 g vs. EC CD36°/LDLR°: 30.17 ± 0.826 g) or females (fl/fl *CD36*/LDLR°: 24.44 ± 0.403 g vs. EC CD36°/LDLR°: 22.5 ± 0.57 g).

Glucose tolerance testing after 10 weeks of diet feeding showed female EC CD36°/LDLR° mice had swifter glucose clearance compared with controls, and both males and females had lower fasting glucose (Figures 7A,B). Body weights measured at the time of glucose tolerance testing did not differ for females (24.4 + 0.434 g vs. 23.6 + 0.51 g, fl/fl *CD36*/LDLR° vs. EC CD36°/LDLR°, respectively), but fl/fl *CD36*/LDLR° males were significantly heavier than EC CD36°/LDLR° males (35 + 0.646 g vs. 30.71 + 1.085 g, p < 0.02). Tracking backward, female mice were not different in terms of clearing a bolus of glucose at baseline (Figure 8A). After 3 weeks of high-fat, high-cholesterol diet feeding, EC CD36°/LDLR° females showed faster glucose clearance, reduced plasma total, and free cholesterol and triacylglycerides (Figures 8B–D) (this was a different cohort). At 8 weeks of diet feeding, EC CD36°/LDLR° females weighed less (Figure 8E). Lipoprotein analysis after 16 weeks showed no differences in male mice (Table 2). Female EC CD36°/LDLR° mice showed a greater percentage of triacylglycerides in the very low density lipoprotein (VLDL) fraction compared with controls (Table 2). Female EC CD36° mice showed less triacylglycerides overall and a delayed increase in plasma cholesterol.

**Table 2:** Lipoprotein Analysis of Mice Fed With the HFHC Diet.

	**fl/fl ***CD36***/ LDLR^°^**	**EC CD36^°^/ LDLR^°^**	**fl/fl ***CD36***/ LDLR^°^**	**EC CD36^°^/ LDLR^°^**
**HFHC Diet**	**Cholesterol (% of total)**		**Triacylglyceride (% of total)**	
**Males**				
VLDL	26	26	62	64
LDL/IDL	47	48	27	29
HDL	27	26	12	7
**Females**				
VLDL	29	23	72	57
LDL/IDL	54	57	18	24
HDL	18	21	11	20

## Discussion

Endothelial CD36 is recognized for its importance in angiogenesis; its role in tissue FA uptake at this interface is less studied. There is now increased awareness that ECs are a second obstacle to FA uptake into tissues (64–66). Previously considered as a passive barrier to FA diffusion, studies have shown regulation at this interface by CD36 and many of the same proteins involved in tissue FA uptake (64–69). CD36 was first implicated in the management of EC FA uptake in research on the EC-specific peroxisome proliferator activated receptor γ° mouse, which has markedly reduced EC CD36 expression and in many ways mimics the metabolic phenotype of the CD36°(70). This implies significant uncompensated metabolic control by EC. Changes in systemic metabolism may partly explain atherosclerosis protection in global CD36° mice, as obesity and insulin-resistance are strong determinants of lesion development.

Conscious of the potential confounding impact of the loss of CD36 in macrophages and other hematopoietic cells on atherosclerosis development and systemic inflammation, we created an EC CD36° using the Tie2e cre, which spares these cells (58–60). Floxed CD36 mice were previously generated utilizing C57Bl/6j embryonic stem cells to avoid background issues (44). These mice, therefore, differ in these ways from previously published EC CD36° mice, which were created using the Tek cre on a mixed genetic background (71). The development of an animal model to study EC CD36 in FA uptake and in the context of a pro-atherosclerotic environment is an important outcome of this work. Additionally, these mice will allow study of EC CD36 signaling pathways, triggered by excess dietary fat, which may also contribute to EC inflammation, plaque development, and pathological metabolic states.

A major finding of this study is that there was a strong contribution of EC CD36 to metabolic homeostasis in both male and female mice, yet there were interesting differences. At 4 weeks of age, when EC CD36° and control mice have similar lean body mass (93–95%) and fat stores, indirect calorimetry showed an increased reliance on carbohydrate metabolism and decreased locomotor activity and energy expenditure in male mice only. Both control and EC CD36° male mice showed mixed carbohydrate and fat utilization during the early light period, based on RER. EC CD36° male mice ate more than controls in the light period, and toward the latter part, showed greater carbohydrate metabolism. During the dark phase, when mice are most active, EC CD36° male mice had decreased locomotor activity; oxygen consumption and carbon dioxide production, which are indicative of energy expenditure, were reduced in both periods compared with controls. This may reflect reduced adenosine triphosphate (ATP) generated as a result of anaerobic respiration of carbohydrates. These data suggest a systemic change in energy metabolism, with a decrease in high energy producing FA oxidation. It will be important to continue these studies in older mice to understand if these early metabolic patterns persist in both sexes, and to determine the effects of different diets.

The metabolic data in males are similar to what has been shown for the global CD36°: increased utilization of carbohydrates over fat as an energy substrate, specifically in heart and muscle (12, 36, 45, 72). Unlike global CD36° mice, the weights were similar between EC CD36° and controls. The data also reveal dichotomy between the sexes that was not observed in the global CD36°. Systemic metabolic changes resulting from reduced uptake of FA were also shown for EC CD36° made with the Tek cre, and they also showed sex-dependent differences: male mice showed a significant decrease in oleic acid uptake into heart, muscle, and brown adipose, while females had no difference in uptake in muscle (71). If true of our mice, this may explain why females displayed no differences by indirect calorimetry. The data may suggest a sex difference in CD36 expression in EC subsets, or a sex difference in expression of alternative transporters. The role of estrogen *vs*. testosterone would be an important area of future study in this regard.

When fed with a low fat (4%), normal chow diet, EC CD36° mice showed faster glucose clearance at 4–5 months of age, while of similar weight. These data are similar to the findings in the global CD36° and EC CD36° made with the Tek cre (36, 71). When the mice were stressed with diets higher in fat than normal chow, weight gain did not differ compared with controls at most time points; females showed transient differences. Female EC CD36° mice continued to show faster clearance of a bolus of glucose in the context of both the 10 and 45 kcal% fat diets. Male EC CD36° mice showed swifter glucose clearance than controls when fed with the 45 kcal% fat diet. In overnutrition, macrophage pro-inflammatory signaling and other adipocyte-derived signals, along with excess FA uptake in muscle, lead to impaired insulin signaling and impaired glucose tolerance (73). Although we have not ruled out differences in insulin secretion, EC CD36° male mice may have increased glucose clearance due to an inability to efficiently utilize FA in heart and muscle and concomitant increased reliance on glucose. This may also be the case in females, if they too have decreased muscle FA uptake. Interestingly, Tek cre EC CD36° females were protected against insulin resistance, despite normal FA uptake in muscle (71). This may suggest that CD36-dependent EC inflammation contributes to systemic inflammatory pathways leading to insulin resistance. This is an important area of further study.

Distribution of cholesterol and triacylglycerides among lipoproteins was not different between male EC CD36° and control mice fed with the 10 kcal% fat diet; female EC CD36° mice showed a 23% enrichment in triacylglycerides in the LDL/IDL fraction. When fed with the 45 kcal% fat diet, both male and female EC CD36° mice showed this shift in triacylglycerides to LDL/IDL (23 and 40% increase for males and females, respectively). These data suggest a delayed ability to hydrolyze triacylglycerol from the VLDL particle. In the global CD36° mouse, increased plasma-free FA were found to inhibit the activity of lipoprotein lipase, leading to decreased VLDL hydrolysis (74). It is possible that the local free FA concentration at the EC interface rises to inhibitory levels, due to loss of EC CD36 and slower tissue uptake, preventing optimal hydrolysis. An alternative hypothesis is that EC CD36 is important in tethering the lipoprotein for lipase activity. Further experiments need to be completed to unravel the mechanism.

As shown, weight gain was similar between the groups on all diets. This was also true of the EC CD36° made with the Tek cre, which showed no difference in white adipose tissue FA uptake (71). In the global CD36° mouse, TG-rich VLDL accumulated, as a result of inefficient hydrolysis both at the EC and tissue surfaces (36). One potential reason why weight may not be affected in the EC CD36° may be that although hydrolysis is inefficient, as the lipoprotein becomes smaller in size, it can enter the subendothelial space and undergo hydrolysis at the level of the individual tissue, and FAs are then taken up by adipocytes for storage.

Activated endothelium provides the surface for initiation of atherosclerotic lesions. We were interested in how atherosclerosis would develop in the absence of EC CD36, because high-fat diets promote atherosclerosis in part through changes in shear stress and other pro-inflammatory pathways. CD36 is an important receptor for pro-atherosclerotic-modified LDL ligands, often called oxPC CD36 (75, 76). These ligands are recognized as a consequence of CD36 scavenger receptor activity. In macrophages, interaction with these ligands leads to the generation of foam cells, reactive oxygen species generation, and secretion of cytokines (46, 75, 76). While EC do not accumulate lipids to form foam cells, we considered that interaction between EC CD36 and oxPC CD36 may contribute to the initiation phase of atherosclerosis through inflammatory pathways. Lesion analysis showed a significant decrease in aortic atherosclerosis burden in EC CD36°/LDLR° female mice. These results differ from global CD36°/LDLR°, where protection against atherosclerosis was observed in both males and females (29).

Endothelial cell CD36°/LDLR° female mice showed no differences in plasma cholesterol levels compared with controls after 16 weeks of diet feeding, and only a transient small difference in weight after 6–8 weeks of diet feeding (but not at 3 or 16 weeks). EC CD36°/LDLR° mice showed decreased fasting glucose, and glucose tolerance testing showed faster glucose clearance in female EC CD36°/LDLR° mice compared with controls. This trait was apparent at the 3 week time point when female EC CD36°/LDLR° mice also showed a decrease in plasma total and free cholesterol and triacylglyceride levels. While improved glucose clearance continued to be a hallmark of EC CD36°/LDLR° female mice, differences in cholesterol and triacylglyceride levels were not found at 6 and 16 weeks of diet feeding. Lipoprotein analysis revealed that EC CD36°/LDLR° female mice had 33% more triaclylglycerides in the LDL/IDL fraction compared with controls after 16 weeks diet feeding. EC CD36°/LDLR° males were similar to controls. The atherogenic diet, although similar in kcal% fat content, differs from the 45 kcal% diet in terms of fat source (milkfat vs. lard) and sucrose amount (341 vs. 206 g/kg). Unlike in the case of the 45 kcal% diet, male EC CD36°/LDLR° mice did not show a difference in glucose tolerance testing, and this may, in part, explain the lack of atherosclerosis protection. Male mice at 4 weeks of age had systemic metabolic changes that suggested greater carbohydrate utilization, and perhaps greater capacity to resist the detrimental effects of excess FA, similar to the global CD36°. The high-fat, high-cholesterol diet, however, may have resulted in a systemic inflammatory response that was greater than in the global CD36° due to CD36 macrophage expression and interaction with CD36 ligands. Further investigation is necessary to understand the interaction of dietary components and sex/sex hormones in the metabolic and atherogenic differences uncovered.

Atherosclerosis is a chronic inflammatory disease that begins in atheroprone regions of the vasculature with activation of the endothelium due to high-fat, cholesterol-rich diets. Inflammation affects the development of obesity and dysfunctional fat, and in muscle, insulin resistance, which are contributing risk factors. EC CD36°/LDLR° female mice showed systemic metabolic differences compared with controls, and had less atherosclerosis lesion burden, but similar weight gain. The inflammatory characteristics of the EC and fat will be interesting to characterize in future studies, as well as analysis of plasma cytokines/chemokines, as these may provide greater mechanistic insight into the protection against lesion development, and the differences between the sexes.

In addition to EC CD36 affecting tissue FA uptake, beyond the scope of this study, there may also be FA effects directly on EC. A recent report by Bou Khzam et al. (77) used a knockdown approach to decrease expression of CD36 by ~50% in mouse lung and cardiomyocyte EC; they found no difference in survival or proliferation. In studies done in the context of added FA (primarily oleic acid), they noted that knockdown of CD36 attenuated FA-induced increases in proliferation and migration in an *in vivo* scratch wound healing assay (77). Palmitic acid had opposite effects and was toxic. Oleic acid-treated, CD36 knockdown EC showed an ~8× increase in AMP-activated protein kinase expression, indicative of EC stress, and a pro-angiogenic response (77). The effect of added oleic acid in the context of reduced but not absent CD36 could perhaps lead to an insulin-resistant state, and similar to in diabetics, inhibits vessel formation, even in the context of pro-angiogenic signals. Using the Tek cre CD36° mice, Bou Khzam et al. (77) showed impaired angiogenesis, accompanied by upregulation of other anti-angiogenic proteins. A previous study measured hindlimb ischemia in global CD36° mice: Isenberg et al. (78) found no difference in limb survival at 7 days between wild type and CD36° mice. They attributed these findings to thrombospondin-1-CD47-dependent nitric oxide antagonism, leading to inhibition of vascular remodeling in this ischemic model. Bou Khzam et al. (77) did not investigate CD47 signaling in their report. Other studies have shown CD36 to be anti-angiogenic *via* thrombospondin-1 signaling, leading to apoptosis in normal EC, or by blocking the vascular endothelial growth factor receptor 2 pathway in tumor EC (79).

How CD36 facilitates FA uptake remains controversial, with at least three different proposed mechanisms. Hamilton and coworkers suggest that CD36 does not play a direct role in the uptake of FA, and flip-flop of FA is the prevailing mechanism (32, 80). Instead, they postulate that CD36-dependent signaling effects FA esterification and incorporation into different cellular pools, and this is why loss of CD36 appears to inhibit FA uptake (35). Based on the structure of CD36, which contains a hydrophobic cleft that reaches near to if not below the lipid bylayer, others have proposed that this acts as a tunnel through which FAs are led to the cell surface, where they may flip-flop over (81, 82). Localization of CD36 to specific plasma membrane microdomains may associate it with cytoplasmic FA binding proteins or esterifying enzymes, leading to apparent unidirectional FA flow (83). Hao et al. (84) provide data in support of a third hypothesis: in the presence of FA, CD36 in adipocyte caveolae is depalmitoylated permitting endocytosis. After delivery of FA to lipid droplets, repalmitoylated CD36 is returned to the membrane surface (84). This mechanism differs from the previous two in the rate of uptake of FA. Whether this pathway extends to all cell types remains to be elucidated. While there may be no consensus in terms of mechanism, it is clear that loss of CD36 in mice and humans leads to systemic effects (11).

Overall, in this report, we show metabolic differences consistent with reduced ability to hydrolyze and uptake FA in the absence of EC CD36, leading to decreased FA oxidation and reduced energy generation. Our work supports a previous study showing EC CD36 is necessary for optimal FA uptake by heart and muscle, and demonstrating changes in glucose tolerance testing, fasting glucose, but not weight, similar to ours (71). We further show that loss of EC CD36 led to reduced aortic atherosclerosis lesion in female mice. To our knowledge, this is the first report of the impact of loss of an EC-specific FA transport protein on atherosclerosis. The data suggest a sex-dependent role for EC CD36 in lipid metabolism, with potential impact on insulin resistance and EC inflammation, driving atherogenesis. These findings support as essential role for the endothelium in controlling tissue FA uptake and for EC CD36 in atherosclerosis development.

## Data Availability Statement

The original contributions presented in the study are included in the article/supplementary material, further inquiries can be directed to the corresponding author.

## Ethics Statement

The animal study was reviewed and approved by the University of Alberta Animal Care and Use Committee.

## Author Contributions

URR helped in the design and planning of experiments and conducted experiments, compiled and analyzed data, and helped write the manuscript. MO helped in the atherosclerosis experiments. MA assisted with animal work. CD designed and carried out the flow cytometry experiments. LI provided assistance with experiments. SE oversaw the flow cytometry experiments. MF helped in the design and planning of experiments and conducted experiments, compiled and analyzed data, and drafted the manuscript. All authors contributed to the article and approved the submitted version.

## Funding

We gratefully acknowledge funding from the Heart and Stroke Foundation (GIA G-17-0019162, MF), Canada Foundation for Innovation, Alberta Enterprise and Advanced Education and the Faculty of Medicine and Dentistry, University of Alberta Motyl Graduate Studentship in Cardiac Sciences (URR).

## Conflict of Interest

The authors declare that the research was conducted in the absence of any commercial or financial relationships that could be construed as a potential conflict of interest.

## Publisher's Note

All claims expressed in this article are solely those of the authors and do not necessarily represent those of their affiliated organizations, or those of the publisher, the editors and the reviewers. Any product that may be evaluated in this article, or claim that may be made by its manufacturer, is not guaranteed or endorsed by the publisher.
